# Annotations

**Published:** 1904-09-17

**Authors:** 


### ANNOTATIONS.

### Medical Men and Hospital Abuse.

The proposal of the London Hospital to inquire into the financial status of out-patients has aroused considerable comment in the Press. There is nothing new after all in the action, except, perhaps, the idea of enlisting the aid of a Charity Organisation officer. But the arrangement has been announced during the silly season and an unusual amount of publicity appears to have been focussed on it. In the present instance the medical interests appear to be most involved. In one interview which was published an official of the Hospital is reported to have intimated that it was not to remedy an abuse which practically was non-existent, but to appease the local practitioners, who felt that the hospital was robbing them of clients. But if this was the intention of the authorities it has signally failed. One member of the profession has already pointed out in the Press that, should a patient prove to be able to pay for medical attendance, the hospital does not refer him back to his medical attendant, but it collects a toll for itself, so that the hospital becomes a self-confessed rival of the private practitioner, and to an extent which, whilst depriving the doctor of fees, adds but little to the hospital funds. And, indeed, the further we go into the matter, the more certain does it appear that the medical profession must suffer pecuniarily, whilst the out-patient department swells its numbers, either upon a whole or partial charity basis. Nevertheless, up to the present time the weight of medical authority has fostered the system, and the number of the pauperised attending the out-patient department of the hospitals has also served as a successful appeal to the public, who hardly wot of the existence of the provident dispensary, many of whose supporters are far more needy than the majority of the less self-respecting recipients of free relief.

### Where Perfection Prevails.

At the recent meeting of the Sanitary Inspectors' Association at Bournemouth, attention was called in the presidential address to what was happily described as the "sanitary primacy" of that pleasant watering place in respect of every condition contributory to the health of mankind. Notwithstanding the presence of invalids, Bournemouth has the lowest death-rate of any "great town" in the United Kingdom, and it has also the lowest death-rate among children under one year of age. It is indebted for these advantages to a general recognition, by the inhabitants, that the prosperity of the town is entirely due to its success as a place of health manufacture, and hence, in order that the progress of this highly useful industry may be uninterrupted, not only have skilled health officers and inspectors been appointed, but every effort has been made to give effect to their warnings and to follow their advice. It is related that Coleridge, when convinced of the desirability of abandoning the vice of intemperance, hired a manservant whose duty it was to restrain his employer, by force if necessary, from entering a public-house, and who was provided with a written authority to this effect. This done, the poet conceived that he had shifted all responsibility for his own sobriety from his own shoulders to those of his attendant, and he deliberately set himself to work to deceive and escape from the man whenever it was possible. On one such occasion he induced the man to climb a tree in search of an imaginary bird's-nest, and, as soon as he had attained a sufficient elevation to be out of the way, set off himself as fast as he could run to a tavern which he knew to be in the vicinity. Bournemouth declines to follow even so distinguished an example, and the tradesmen who are the most frequent creators of nuisances flock, so it appears, to the sanitary inspector, and beg of him to call upon them to do something which may at least suffice to display their earnestness. Piggeries vanish at a suggestion, and no objectionable condition in any eating-house or restaurant has been able to survive a single gentle admonition. Dairymen have not only framed rules by which to restrain each other within the strictest paths of virtue, but they have met in conclave to require that the rules should be extended to cover the sources of milk supply in the vicinity. Magistrates, in relation to sanitary matters, have become the merest superfluities. Indeed we cannot resist the belief that, were the example of Bournemouth followed by other centres of population, we should be brought appreciably nearer to the millennium.

### Insanity Statistics.

The annual report of the Commissioners in Lunacy which has just been issued, states that the number of persons living in England and Wales on January 1st, 1904, certified as insane was 177,199, these figures showing an increase of 3,235 over the corresponding date of the previous year. Compared with the increase of the population during the decade ending in 1901, which is estimated at 12·2 per cent., the increase of the insane was 24·4 per cent. The usual reports in reference to the "causes" of insanity are given by the Commissioners, but they do not show any particularly novel feature; alcoholic intemperance takes a prominent place, 22·8 per cent. of the male cases and 9·5 per cent. of the female cases being attributed to this cause. In general paralysis and dementia the male sufferers are in excess of the females, whilst the reverse is the case in mania and melancholia. For some years all alleged cases of lunacy in paupers in Glasgow have been referred to Mr. Carswell, and he has had an opportunity to detain in a special ward all instances of what he considered mere temporary mental disorder. As a result, a very large percentage of these patients have recovered without being sent to an asylum. Thus, out of 261 admissions to the observation wards during last year, 24 were removed to an asylum, 13 died, and the remaining 219 were discharged to the privileges of civil freedom. These results are obviously of great importance and justify a large extension of the scheme which is under Mr. Carswell's direction. This extension is to take the form of a hospital, and thus it is hoped that many cases will be sent in their early stages and when the chance of a cure is correspondingly great. It is the dread of the asylum in the popular mind which prevents the opportunity of prompt and successful treatment.

